# Lymphedema: current treatment strategies and future directions in tissue engineering

**DOI:** 10.3389/fmed.2026.1851346

**Published:** 2026-08-05

**Authors:** Alina Stoian, Mariana Jian, Angela Babuci, Vitalie Cobzac, Ana-Maria Nacu, Mihaela Sîromeatnicov, Dumitru Hîncota, Nicolae Caproş, Grigore Verega, Viorel Nacu

**Affiliations:** 1 Clinic of Plastic, Reconstructive Surgery, and Microsurgery of the Locomotor System, Department of Orthopedics and Traumatology, Nicolae Testemitanu State University of Medicine and Pharmacy, Chişinău, Moldova; 2 Laboratory of Tissue Engineering and Cellular Culture, Nicolae Testemitanu State University of Medicine and Pharmacy, Chişinău, Moldova; 3 Department of Orthopedics and Traumatology, Emergency Medicine Institute, Chişinău, Moldova; 4 Department of Anatomy and Clinical Anatomy, Nicolae Testemitanu State University of Medicine and Pharmacy, Chişinău, Moldova; 5 Department of Orthopedics and Traumatology, Nicolae Testemitanu State University of Medicine and Pharmacy, Chişinău, Moldova; 6 Department of Clinical Anatomy and Operative Surgery, Nicolae Testemitanu State University of Medicine and Pharmacy, Chişinău, Moldova

**Keywords:** lymph node, lymphatic tissue engineering, lymphatic vessels, lymphedema, tissue engineering

## Abstract

The lymphatic system is a part of the circulatory system, playing a crucial role in fluid balance and immune function. Dysfunction of the lymphatic system can lead to lymphedema, a chronic and progressive condition characterized by the accumulation of lymphatic fluid, resulting in tissue swelling, pain, and functional impairment. Lymphedema is classified as either primary or secondary. Primary lymphedema arises from congenital dysfunction of the lymphatic system and may be associated with various genetic syndromes or occur idiopathically. Secondary lymphedema is associated with damage, obstruction, or removal of previously normal lymphatic vessels or lymph nodes. Traditionally, management strategies for lymphedema have been predominantly conservative, and even now, conservative treatment with complete decongestive therapy remains the gold standard. However, in recent years, lymphatic surgical approaches such as lymphaticovenous anastomosis and vascularized lymph node transfer have been improved by advances in microsurgical techniques, with promising clinical outcomes. Equally important, lymphatic tissue engineering has emerged as a promising new direction in lymphedema treatment, introducing both new challenges and opportunities. The complex tissue and molecular alterations associated with lymphedema remain major obstacles to developing a universally applicable treatment algorithm. As no curative treatment is currently available, this review aims to provide a comprehensive overview of current therapeutic options for lymphedema, highlight key experimental advances in lymphatic tissue engineering, and discuss their potential translational applications.

## 1 Introduction

Lymphedema (LE) is a complex and progressive disease that occurs as either a primary or secondary condition. While primary LE is a rare diagnosis, the incidence of secondary LE continues to increase worldwide in parallel with the global rise in cancer incidence (1–3). By medical definition, LE can be described as a localized form of tissue swelling and fibroadipose tissue deposition due to dysfunction of the lymphatic vessels (LVs) or lymph nodes (LNs). This dysfunction may be congenital or associated with an underlying systemic disease, trauma, or surgery (4–7). In addition to edema, clinical signs of LE include skin changes, which are correlated with disease stage and/or lack of treatment, leading to potential cutaneous sequelae (7, 8). The accumulation of protein-rich lymph in the interstitial space triggers a chronic inflammatory response, leading to progressive fibrosis, pathological adipose tissue deposition, impaired wound healing, and immune dysfunction. The mechanisms underlying LE-associated adipose tissue expansion remain incompletely understood (9).

For a long time, complete decongestive therapy (CDT) was considered the only treatment option for both types of LE. In recent years, lymphatic surgical approaches such as lymphaticovenous anastomosis (LVA) and vascularized lymph node transfer (VLNT) have been revolutionized by advances in microsurgical techniques, demonstrating promising clinical outcomes. However, the limitations of this technique, especially in VLNT, including donor-site morbidity, highlighted the need to expand therapeutic options (10). In this context, a tool with potential for future applications, including tissue engineering (TE), is under exploration.

Regarding progress in LE management, TE has the potential to offer new opportunities for treatment, offering a new approach that targets the underlying cause of the disease rather than merely addressing its progression and/or complications. By promoting regeneration through the development of new lymphatic tissue using cells, growth factors (GFs), biomaterials, scaffolds, and *in vitro* and *in vivo* models (11–13).

This review article summarizes current treatment methods for LE, provides basic clinical and physiological descriptions of the disease, and highlights emerging directions in lymphatic TE (LTE), including key experimental breakthroughs and their potential translational applications. Article selection was conducted using PubMed, Google Scholar, and Scopus, with a time restriction from 2000 to the present. Studies were first screened based on title and abstract, followed by full-text assessment of potentially relevant articles. The search strategy combined keywords related to LE, LTE, lymphangiogenesis, scaffolds, cells, GFs, and treatment options. Additionally, the reference lists of all eligible studies were manually screened to identify further relevant articles not retrieved in the initial database search. The literature was collected, organized, and managed using Paperpile reference management software. A total of 387 articles were retrieved from PubMed, Scopus, and Google Scholar. After title and abstract screening, 147 articles were excluded. The remaining 240 full-text articles were assessed for eligibility, and 86 were excluded based on predefined inclusion and exclusion criteria. In addition, the reference lists of eligible studies were manually screened, identifying additional relevant articles. Ultimately, 177 articles were included in the final review. No duplicate articles were identified, as Paperpile automatically indicates whether a record has already been added upon import into the library. Table 1 (Supplementary material) details the full article selection process.

**TABLE 1:** Factors regulating lymphatic flow (3).

Factor types	Example
Extrinsic	- Muscle activity;
	- Heart contraction;
	- Respiration.
Intrinsic	- Peristaltic and segmented contractions of lymphangions;
	- Lymphatic valves.
Others	- Humoral stimuli;
	- Neuronal stimuli;
	- Mechanical stimuli (pressure and shear stress);
	- Flow-mediated nitric oxide production by endothelial cells, determining dilation of vessels;
	- Increased microvascular permeability under the action of high concentrations of atrial and brain natriuretic peptides.

## 2 Anatomy

**FIGURE 1:**
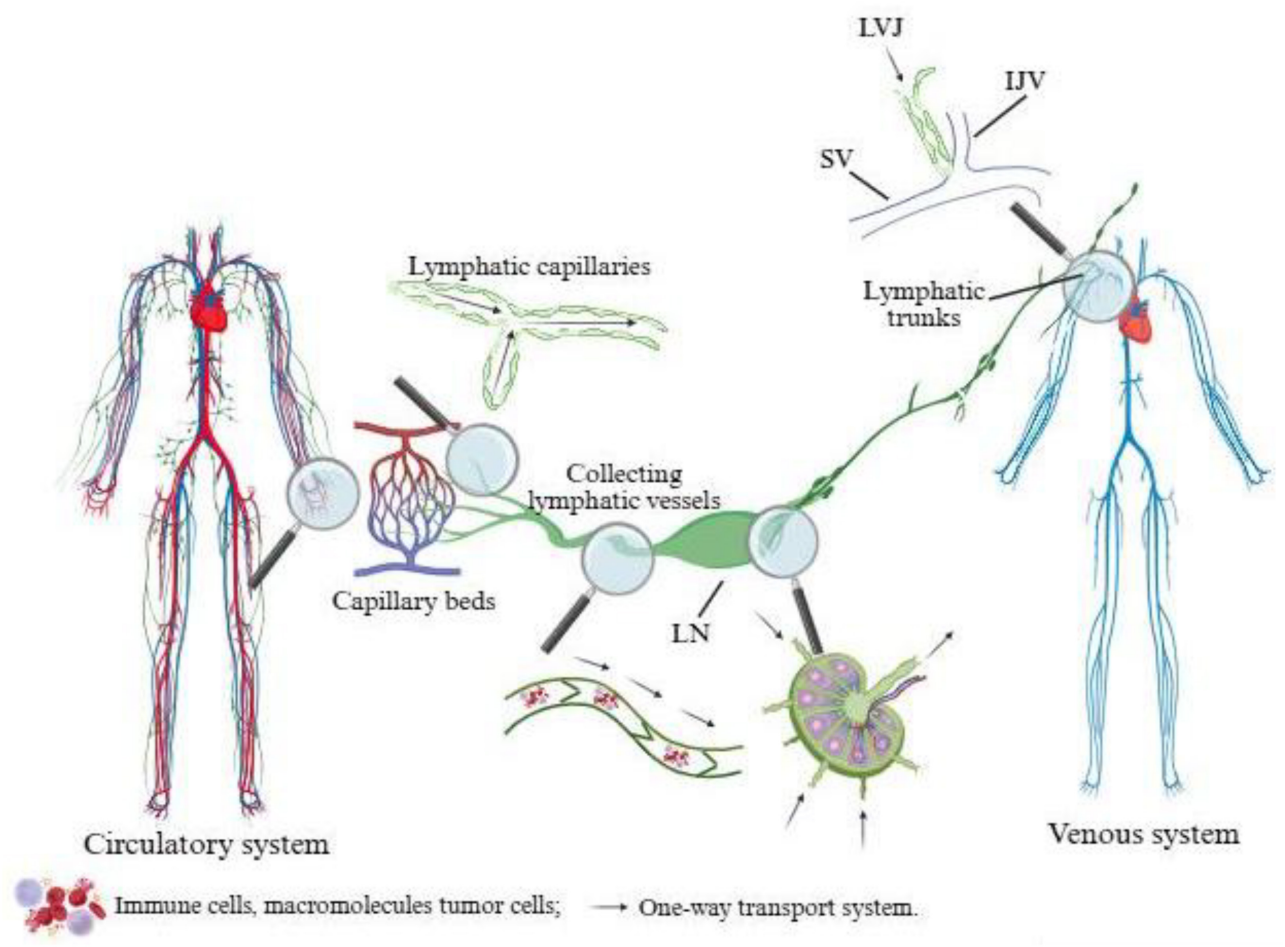
The vascular tree of the human lymphatic system (21–26). LVJ, lymphovenous junction; IJV, internal jugular vein; EJV, external jugular vein; SCV, subclavian vein; LN, lymph node.

The lymphatic system (LS) is a part of the circulatory system, which consists of complex macro- and microscopic morphological structures such as primary and secondary lymphoid organs. The primary lymphoid organs. include the red bone marrow and thymus (14, 15), and the secondary lymphoid organs are the spleen, regional LNs, aggregated lymphoid nodules of ileum and vermiform appendix, and pharyngeal lymphoid ring (Nasal Associated Lymphoid Tissue) (3, 16, 17). The vascular components of the LS consist of lymphatic capillaries (initial lymphatics characterized by thin walls, blind-ended structure, high permeability, and absence of valves), collecting LVs (pre- and post-nodal), and lymphatic trunks (Figure 1) (15, 18, 19).

The lymphatics represent heterogeneous vessel networks that comprise a one-way transport system through the body, regulating tissue fluid balance and transporting immune cells as well as tumor cells, along with interstitial macromolecules (15, 20). Through the afferent vessels, the migration of lymph toward the LNs is ensured, and via the efferent vessels, the filtered lymph drains into larger LVs and lymph trunks (lumbar, intestinal, bronchomediastinal, subclavian, and jugular), and finally into the right lymphatic duct and the thoracic duct. The right lymphatic duct drains into the right venous angle, and the thoracic duct opens into the left venous angle (3, 14).

In addition to the main function of the LVs, the LS plays a crucial role in the formation and function of specific organs (27, 28). LS function is required to prevent adipogenesis and obesity (29–31), contributes to cardiac cell regeneration (32, 33), allows alveolar maturation in the newborn, and regulates adult lung homeostasis, being a key component in the pathogenesis of respiratory diseases (28, 34). Furthermore, meningeal lymphatics remove damaged proteins from the brain, and impairment of this system plays a direct role in Alzheimer’s disease (35, 36).

For a long time, the LS was considered a secondary vascular system, and its disorders were mainly associated with LE. More recently, specific endothelial cell (ECs) markers of LVs, distinct from those of blood vessels, have been identified, and the LS has become recognized over time as a valuable part of the vascular system, no longer secondary to the blood vascular system (27, 37, 38).

Considering the complexity of the morphological components of the LS, LE can be defined as a complex and progressive pathologic condition, characterized by disturbances of both lymphatic and venous systems. In LE, due to damage and/or obstruction of the LVs, an impaired drainage of the rich-protein interstitial fluid occurs (39, 40). A dilation of the collecting vessels, followed by hyperplasia of the smooth muscles and lack of lymphatics contraction, leading to reduced lymph flow and lymphostasis, with subsequent perilymphatic inflammation, tissue remodeling, fibrosis and impaired adipogenesis (41–43). Unfortunately, there still persists some unclearness regarding the time sequences of the pathogenetic processes (16).

Lymphatic flow is regulated by pump mechanisms influenced by extrinsic and intrinsic factors (Table 1) (3). A key feature enabling lymphatics to function is their permeability, which was long thought to be absent. Recent studies have shown that LVs are permeable, and this property can be modulated by pathological conditions (44), or by peptides such as atrial and brain natriuretic peptides (45).

## 3 Etiopathogenesis and diagnosis

LE is classified as primary LE or secondary LE. Primary LE is caused by a congenital dysfunction of the LS, can be associated with various genetic syndromes or can be idiopathic. The diagnosis of primary LE can be difficult or delayed (4, 40, 46). Depending on age, there are three main groups of primary LE: (1) lymphedema congenitalis in patients younger than 1 year of age; (2) lymphedema praecox in patients aged 1–35 years; (3) lymphedema tarda in patients older than 35 years. The genetic disturbances are influenced by over 20 various genes. In VEGF-C and VEGFR-3 mutations a regional or generalized form of LE is installed, while in mutation of CCBE-1 and ADAMTS-3 the LE might be widespread or local, implying intestinal lymphangiectasia and other mesenteric anomalies. The mutations in EPHB-4, FOXC-2, GATA-2, PIEZO-1, GJC2 and FAT4 genes, encode proteins responsible for maturation and remodeling of the lymphatic circulation at the initial stages of embryonic development (2, 3). Lymphatics malformations can also lead to primary LE (47). The abnormalities are characterized by lesions of the endothelial lining on the LVs, dilated lymphatics with macro-, micro and mixed cystic-like areas filled with lymph, preponderantly located in the head and neck, axillary and pelvic regions (3). In mutations of the VEGF-C gene or of its receptor (VEGFR-3) (43), and in functional disturbances of the shear stress signaling pathways, abnormal vasculogenesis (28), with hyperplasia, hypoplasia or aplasia of the LVs occurs (48).

On the other hand, secondary LE is associated with damage, obstruction, or removal of previously normal LVs or LNs, and represents a common side effect of oncological treatments. Its diagnosis and progression depend on the extent of lymphatic involvement. The condition may be asymptomatic in certain patients, yet advance quickly in others (49, 50). Breast cancer-related LE generally develops about 8 months after surgery, with 80% of cases diagnosed within the first 3 years (51). Lower-extremity LE following pelvic or inguinal LNs dissection typically develops more rapidly, within 3–6 months postoperatively (52). According to Lee and Kim (42), there are three main mechanisms of SLE development (Table 2).

**TABLE 2:** Key mechanisms in secondary lymphedema.

Mechanism	Description
Inflammation	- Implying activation of the CD4 + T cells;
	- Influencing the VEGF-C and VEGFR-3;
	- Contributing to abnormal lymphangiogenesis.
Impaired adipogenesis	- With adipocyte hypertrophy and lipid accumulation, which occur in response to lymphostasis, there is increased expression of the key transcription factors CCAAT/enhancer-binding protein α (C/EBPα) and peroxisome proliferator-activated receptor-γ (PPARγ), responsible for adipocyte differentiation and proliferation.
Tissue fibrosis	- Under hyperactivity of Th2 cells, inducing secretion of profibrotic cytokines IL-4, IL-13 and of the growth factor TGF-β1.

**FIGURE 2:**
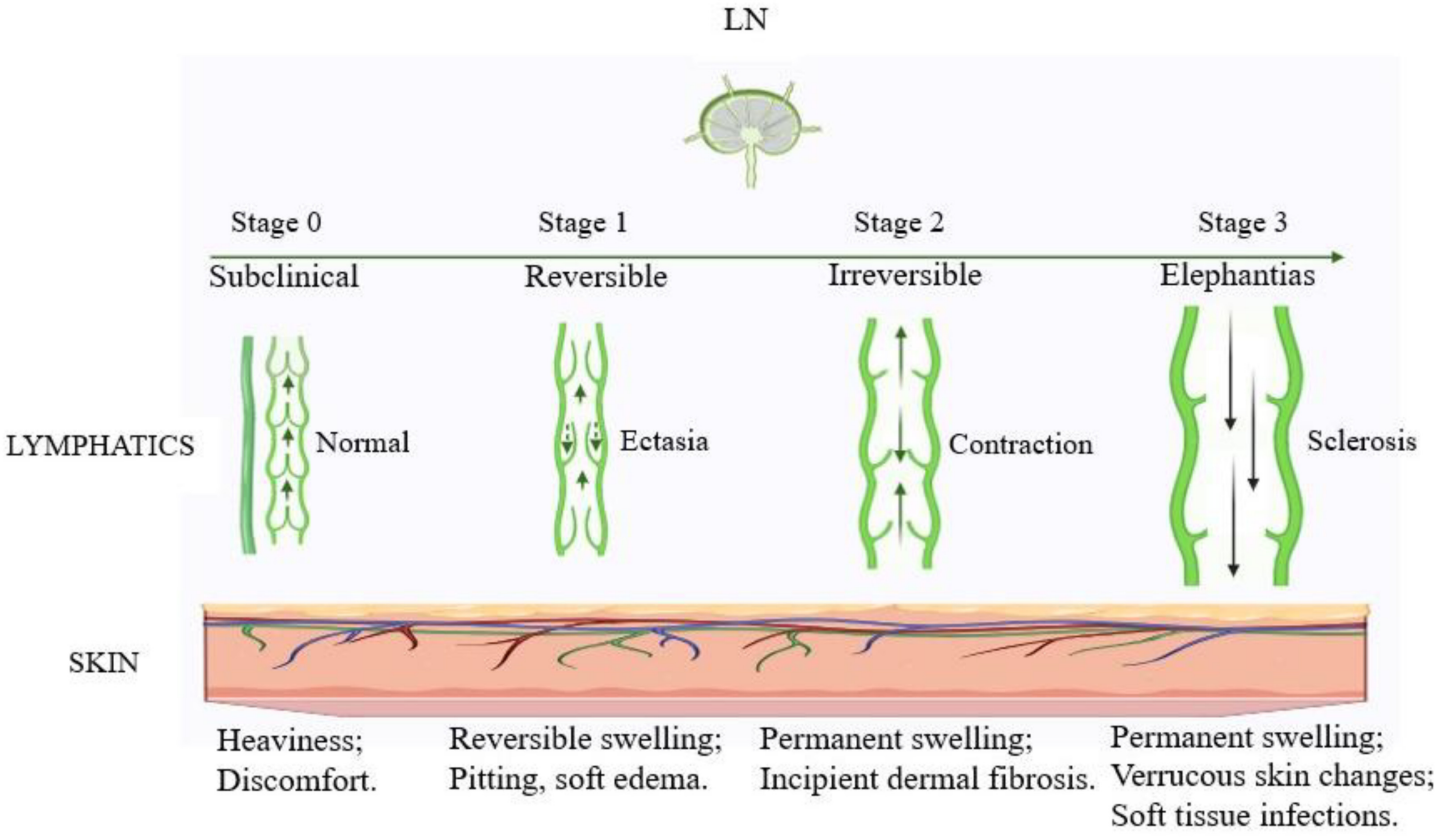
Staging of lymphedema (56–58).

Among the risk factors of secondary LE should be specified the regional LNs dissection, with an incidence in breast cancer ranging. The risk of LE is high, even in sentinel LN biopsy. The radiation therapy, obesity, skin resection, iatrogenic injuries and infections, chronic venous insufficiency were emphasized as risk factors. The highest ratio of LE was reported for sarcoma, followed by gynecologic malignancies, melanoma, tumors of the genitals and urinary systems and the lowest ratio was determined in head and neck cancer (40, 42, 48, 53). There are three forms of head and neck cancer-related LE, identified in over 90% of patients: external (e.g., involving the face and neck), which includes four stages ranging from stage 0 (local swelling that does not affect regular function) to stage 3 (severe swelling, ulcerations, and ability to eat is severely affected); internal (e.g., involving the larynx and pharynx), which can also be measured using a scale and graded from no swelling (0) to severe (3); and combined (54, 55). According to the International Society of Lymphology, secondary LE is classified into four clinical stages (Stages 0–III), which reflect the progressive severity of the disease depending on changes in the lymphatics, as illustrated in Figure 2 (56).

In situations where the diagnosis of LE is not clear from the history and physical examination, imaging studies are used to determine the appropriate method of treatment. Imaging plays a central role in the diagnosis, staging, and treatment planning of LE, providing complementary information on lymphatic anatomy and function. The main characteristics, clinical roles, advantages, and limitations of these techniques are summarized in Table 3.

**TABLE 3:** Lymphedema imaging overview.

Method	Findings	Properties		Role in treatment planning
		Advantages	Limitations	
Lymphangioscintigraphy (LAS)/Lymphoscintigraphy/ Isotope scintigraphy (59–63)	Gold standard for lymphatic imaging; 96% sensitivity and 100% specificity; Illustrate the pathways of lymphatic drainage from the skin to the LNs.	Whole body imaging; Follow-up evaluation after medical treatment.	Exposure to radiation; Expensive; Poor image resolution; Does not assess lymphovenous bypass function.	Differentiate LE from edema of other origins; Assessment of the prognostic of CDT; Assessment of donor-site morbidity after VLN harvest.
Indocyanine green (ICG) lymphangiography/Near-Infrared Lymphography (NIL) (63–67)	Visualizes the pathways of superficial lymphatics and dermal backflow.	Used for precise staging of BC-related LE; No risk of radiation exposure easy to perform.	Needs specialized imaging systems.	Can identify early stages of LE; Real-time investigation of lymphatics; Used for preoperative assessment and postoperative follow-up; Assesses lymphatic flow in free flaps and VLNT; Assessment of LVA function.
Magnetic resonance (MR) lymphography (63, 68–70)	Imaging of lymphatics and tissue anatomy; Can be done with or without contrast injection; Assess the severity of soft tissue edema, subcutaneous fat hypertrophy, and lymphatic channels; Contrast-enhanced imaging allows visualization of dilated lymphatics and dermal backflow.	Improved depth imaging (*vs* LAS and ICG); Provides high-resolution 3D imaging.	Limitation due to the absence of lymphatic-specific tracers; Venous uptake of contrast can impair the imaging; Expensive.	Differentiate LE from edema of venous etiology; Diagnostic evaluation; Used to guide interventions for primary LE involving central conducting channels.
Ultrasound (USG) and Ultra-High frequency ultrasound (UHFUS) imaging (58, 69, 71, 72)	Detecting the LVs and their degeneration status (wall and lumen characteristics).	Non-invasive imaging; Inexpensive.	Limited depth of assessment; USG has limited applicability in monitoring the progression of LE.	UHFUS is used in preoperative planning of LVA with mapping of lymphatics and recipient venules; USG can be used to differentiate LE from lipedema; Can help in the identification of causes such as obstruction and tumors.

LNs, lymph nodes; LE, lymphedema; LVA, lymphovenous anastomosis; VLNT, vascularized lymph node transfer; CDT, complete decongestive therapy.

Preoperative assessment of lymphatic anatomy and function using imaging techniques is essential for selecting the most appropriate treatment (73). ICG lymphography and radionuclide lymphoscintigraphy are essential for surgical planning of the LVA and VLNT. ICG lymphography helps identify the location, course, and flow of superficial lymphatic channels, as well as those up to 2 cm deep. Findings can be categorized as linear patterns, corresponding to normally functioning lymphatic channels and typically seen in patients with mild-stage LE, or dermal backflow patterns, which are characteristic of more severe cases of LE. Dermal backflow patterns correlate with disease severity and fibrosis of the lymphatic channels and can be further classified into splash, stardust, and diffuse patterns. On the other hand, lymphoscintigraphy enables visualization of the number and size of LVs and LNs and facilitates the assessment of dermal backflow. Lymphatic dysfunction is characterized by delayed, asymmetric, or absent visualization (74). Candidates for LVA are patients with stage I or II LE (International Society of Lymphology classification) who have shown minimal improvement with conservative treatment. In these patients, ICG lymphography is performed preoperatively to identify suitable functioning lymphatic channels and guide incision placement (64, 75). VLNT should be considered in patients with significant dermal backflow and no functioning LVs on imaging, in those with stage II LE (International Society of Lymphology classification), and in those who have not improved after at least 1 year of conservative treatment (76, 77).

Overall, the management of LE requires a holistic approach that addresses the disease’s physical, functional, and psychosocial aspects, along with appropriate clinical evaluation and staging (58).

## 4 Current treatments in lymphedema

### 4.1 Conservative treatment

CDT is considered the gold standard for non-surgical treatment of moderate-to-severe LE. CDT includes manual lymphatic drainage, multi-layer compression wraps, and therapeutic exercises, which are often performed in conjunction with compression, skin care, and patient education. CDT consists of two phases: an intensive phase, which aims to reduce limb volume, and a maintenance phase, which aims to preserve the gains achieved during the first phase. The main goal is to mobilize lymph and dissipate fibrosclerotic tissue. In the second phase, the focus shifts to maintenance therapy, with patients encouraged to wear custom-made garments for continuous compression (78). Compression therapy involves applying external pressure to the affected limb to reduce swelling, facilitate fluid movement, prevent fluid reaccumulation, and assists muscle pumping. Compression garments should be used properly to avoid skin irritation and potential risk of infection (79). Physical activity is also a key component of LE prevention, as it activates musculoskeletal pumping, increases venous and lymphatic flow, and promotes lymph drainage and protein absorption through muscle contraction. Aquatic activities are recommended as well (48, 80). Recently, intermittent pneumatic compression has been used in addition to CDT. Although it has not shown extensive positive effects on limb volume, pain, or heaviness, it appears to have a greater beneficial effect on improving external rotation joint mobility (81).

### 4.2 Surgical treatment

In addition to conservative therapy, surgical techniques for LE have become increasingly utilized in recent years (82). As LE is a disease with no curative treatment, surgical interventions can be grouped into two categories: (i) physiologic procedures, which are preferred in the early stages of the disease and aim to restore lymphatic function, and (ii) reductive procedures, which are reserved for moderate to advanced stages and focus on excising affected tissue (50).

Surgical procedures for LE management date back several decades. The concept of surgical treatment is simple: to restore fluid flow through the lymphatics to the peripheral veins. Between 1960 and 1970, due to technical limitations, lymphovenous shunts or coaptation of the LNs stump onto a large vein were performed. Over time, advancements in microsurgical techniques led to the development of microsurgical LVA. Nowadays, the concept of supermicrosurgical anastomosis has gained popularity, meaning the anastomosis is performed on extremely small vessels (0.3–0.8 mm in diameter). These procedures are now preferred, particularly in patients with early-stage LE (83, 84). Together with LVA, VLNT are two surgical techniques used to address LE by reconstructing the LS. VLNT refers to the transplantation of healthy LNs and surrounding tissue. There are two theories explaining how VLNT improves drainage: (i) transplanted LNs act as a new area of low pressure, collecting lymph and transferring it into the venous system; (ii) transplanted healthy lymphatic tissue promotes the spontaneous development of new afferent and efferent lymphatic connections between the donor flap and the native tissues, creating new channels for lymph drainage. This lymphangiogenesis is believed to be promoted by VEGF-C (83, 85, 86). The systematic review by Meuli et al. (87), provides high-quality evidence based on 150 studies (6,496 patients) supporting LVA and VLNT as effective treatments to reduce the severity of secondary LE. The meta-analysis reported outcomes such as decreases in limb circumference (67%), limb volume (36%), and the number of cutaneous infections (35%) (87). Both LVA and VLNT have shown efficacy in primary LE as well (46). Reductive procedures, such as liposuction and excisional techniques, have also been used in patients with LE to achieve positive outcomes in terms of improving quality of life through volume reduction and infection control (88). Used alone or in combination with supermicrosurgical techniques, liposuction can provide more durable and comprehensive outcomes for moderate-to-late stage secondary LE and can improve therapeutic options for patients (89).

LE management includes both conservative and surgical approaches, with treatment selection largely determined by disease stage and response to conservative therapy. Conservative treatment remains the foundation of management across all stages, whereas surgical interventions are generally reserved for patients with persistent symptoms. The main treatment options, their mechanisms of action, clinical outcomes, and limitations are summarized in Table 4.

**TABLE 4:** Conservative and surgical management options for lymphedema.

Treatment method	Indication (Stage)	Therapeutic mechanism	Clinical outcomes	Limitations
Non-surgical methods				
MLD (90–92)	Most effective in early stages; Applicable across all stages.	Stimulates the lymphatics, promotes drainage of excess fluid, and reduces swelling in affected areas.	The studies show significant reduction in limb volume and improvements in symptoms following a MLD treatment, sustaining the therapeutic benefits.	Often used as part of a complex treatment approach; Solely use has less promising results; Limited effectiveness for patients with extensive fibrosis; Limited outcome in obese patients. The effectiveness also depend on the condition of the skin, e.g., Thickened skin interferes with the efficiency due to reduced ability of LVs to respond to external pressure. Patients can develop skin irritation due to excessive pressure.
CT (93–95)	All stages	CT is achieved by applying compression garments, bandages and/or pneumatic compression devices that act on LVs with external pressure.	Evidence suggests significantly reduced limb volume and improved symptoms compared to no treatment.	Garments should be tailored to the patient and properly monitored. Effectiveness is limited in patients with extensive fibrosis or obesity. Thickened skin may reduce lymphatic responsiveness, and issues can include skin irritation, discomfort, and restricted mobility.
Exercises (93, 96, 97)	All stages	Specific exercises can promote lymphatic flow, enhance muscle pumping, reduce swelling, and improve limb function and quality of life. Activities such as walking, swimming, cycling, and aquatic exercises increase range of motion and flexibility. Resistance training may be beneficial but should be done cautiously under supervision.	Low-impact aerobic exercises reduce swelling, improve range of motion and promote cardiovascular health while contributing in weight management.	Require guidance on safe and appropriate activities based on individual needs and health status.
PCT (93, 98–100)	Applicable across all stages.	Therapeutic effect is obtained by pneumatic compression devices that inflate/deflate boots or sleeves attached on the affected limb, promoting lymphatic drainage. PTC works by accelerating tissue fluid movement by mimicking the natural pumping action of muscle.	Effective especially in lower limb edema, by reducing limb volume and improving overall symptoms. PCT is recommended in clinical practice guidelines.	Discomfort in device usage with limited effectiveness in advanced fibrosis.
Surgical methods				
LVA (83, 101)	Early-stage LE; Immediately following axillary LN dissection.	Direct connection of LVs to venules or small veins creating drainage pathways using microsurgical sutures	Reductions in limb circumference; Minimal risk; Minimally invasive procedure; Can be performed under local anesthesia; Reduced frequency of cellulitis; Improvements in the quality of life	Better outcomes in early-stage LE.
VLNT (83, 101)	Advanced stages of LE	Transplantation of healthy LN and surrounding tissue creating drainage pathways, restores immune function, and promotes lymphangiogenesis.	Can provide long-term results; Reduction in LE symptoms; Improved upper limb function; Enhanced body image and improved quality of life.	Risk of complications.
Liposuction (102, 103)	Controversy regarding late-stage LE; Not indicated for patients with pitting edema.	Hypertrophied adipose tissue is removed through short incisions and specific cannulas.	Long-term results have not shown any recurrence of arm swelling, unlike conservative treatment and microsurgical reconstructions, which cannot achieve this.	Contraindicated in patients with active cancer or those medically unfit for surgery; Success requires strict pre- and postoperative compliance with compression garments; Can lead to paresthesia of the skin for 3 to 6 months postoperatively.
Excisional techniques (104)	Used in selected cases with overhanging skin changes or limited treatment resources.	Skin and subcutaneous tissue resection followed by skin grafting.	Significant volume reduction.	Risks of graft failure, delayed healing, infection, hematoma extensive scarring and sensory changes.

MLD, manual lymphatic drainage; CT, compression therapy; PCT, pneumatic compression therapy; CDT, complete decongestive therapy; LVA, lymphovenous anastomosis, VLNT, vascularized lymph node transfer.

### 4.3 Pathophysiology-based emerging therapies

LE is a disease, not merely a symptom, and therapy should be guided by its pathophysiology. Advances in microcirculation and molecular biology have improved the understanding of the morphological and functional tissue changes resulting from lymphostasis. The goal of pathophysiology-based emerging therapies for LE is to restore function, reduce physical and psychological suffering, and prevent the development of infection. Historically, the treatment of LE was based on the regeneration of LVs using cytokines, as it was believed that the disease was caused by surgery-induced damage to LVs. New approaches have increasingly focused, besides lymphangiogenesis, also on targeting chronic inflammatory responses to decrease fibrosis, reduce lymphatic leakiness, and enhance lymphatic pumping (40, 105). Therefore, the primary therapeutic approaches for LE nowadays are more complex and are based on multiple strategies: lymphangiogenic interventions; anti-inflammatory and anti-fibrotic treatments; as well as emerging strategies such as benzopyrones, retinoid-like compounds, topical skin applications, antibiotics, platelet-rich plasma, and stem cell therapies (106–108). However, it is important to note that, even today, pharmacological therapies for LE remain largely at the preclinical stage. Treatments such as 5-lipoxygenase-targeting agents, antifibrotic medications, and tacrolimus have shown promising results in murine models, including improvements in lymphatic flow, reductions in edema and inflammation, and attenuation of fibrosis (109). While most of these approaches have not yet reached routine clinical use, anti-inflammatory therapies have shown potential benefits in clinical trials of patients with LE (110). The potential use of autologous tissues as a treatment for LE has also been evaluated. In a systematic review, Forte et al. (111) evaluated the use of autologous blood components to treat LE. They concluded that blood component–based therapies, particularly lymphocytes and platelet-rich plasma, are promising targeted approaches for LE management. However, the limited number of studies and methodological constraints in the available clinical evidence underscore the need for further research to clarify their efficacy and therapeutic potential (111).

Also, the morbidity of LE may be related to the patient’s BMI. Therefore, an active lifestyle with a normal BMI tends to predict less severe disease compared with individuals who are obese (112, 113). Recent studies indicate that weight loss reduces limb circumference among overweight breast cancer survivors with LE, and a ≥5% reduction has been proposed as a relevant therapeutic target for future management strategies (114).

## 5 Lymphatic tissue engineering

Lymphatic TE uses scaffolds, cells, and GFs to regenerate tissue damaged by disease or trauma. In addition, it enables the development of *in vitro* tissue models to study lymphatic tissue function and *in vivo* models to evaluate disease progression, lymphatic regeneration, and the therapeutic potential of engineered tissues (115, 116). Lymphatic TE is considered a relatively young field because lymphatic-specific markers were discovered less than 30 years ago. At that point, the distinction between the vascular and LSs was established, enabling specific research efforts into LS regeneration. Advances in this field could benefit multiple areas of medicine. Engineered lymphatic tissues and models may improve the study and treatment of cancer metastasis, cardiovascular disease, metabolic disorders, wound healing, and neurological diseases. In addition, they provide valuable platforms for investigating disease mechanisms, evaluating drug responses, developing regenerative therapies and cellular pathways to enhance cancer vaccine delivery to LN (12, 117–119).

However, while the advances made in the study of lymphatic TE are encouraging, the complexity of the LS makes the engineering of functional lymphatic tissues challenging. Key obstacles in lymphatic TE include the selection of appropriate scaffolds, efficient cell harvesting, and ensuring adequate nutrient supply. Despite promising findings from preliminary studies, the recreation of secondary lymphoid organs has achieved more limited success than other areas of TE, largely because of their intricate anatomy.

One of the greatest challenges in LVs engineering is the development of functional valves that can respond appropriately to lymph flow while preventing retrograde flow. Similarly, LN-focused TE must address not only the recreation of structural features but also the replication of their complex biological functions (10, 120, 121). However, advances in this field suggest the possibility of engineering LNs and LVs, which are the most studied components of the secondary LS in relation to the etiopathogenesis of LE (10, 122).

Another important direction in LTE is lymphangiogenesis, in which the availability of lymphatic endothelial cells (LECs) represents a key limiting factor. Thus, in addition to *in vitro* approaches for constructing lymphatic structures, *in situ* regeneration of damaged lymphatics has also gained increasing interest (117, 123).

The following sections address current directions in lymphatic TE relevant to LE, including scaffold-based strategies and enhancement techniques, LNs-focused TE, and approaches to lymphangiogenesis. *In vivo* and *in vitro* studies are also summarized, and their translational potential for LE treatment is discussed in a separate section.

**FIGURE 3:**
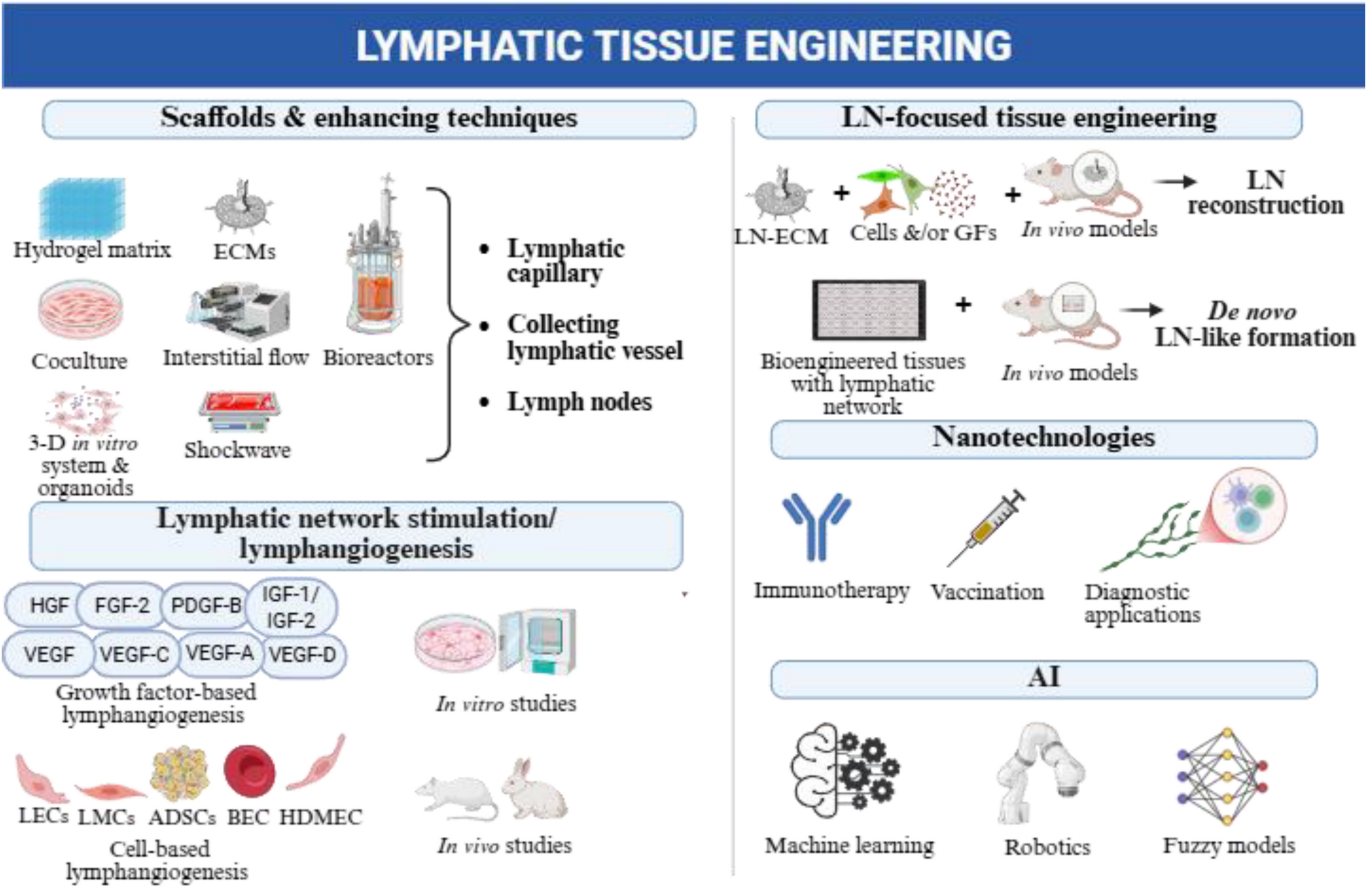
Conceptual framework of lymphatic tissue engineering. The figure provides a clear overview of lymphatic TE, organized into key thematic areas. It emphasizes scaffold-based and biomaterial approaches, stimulation of lymphatic networks via GFs and cellular techniques, applications focused on LNs, and emerging technologies such as nanotechnology and AI. AI-assisted approaches can support biomaterial design, image analysis, and optimize lymphatic TE strategies. Together, these components illustrate the multidisciplinary nature and interconnections of current strategies in the field (11, 12, 115, 118, 123–131). LN, lymph node; VEGF, vascular endothelial growth factor; LECs, endothelial cells; LMCs, muscle cells; ADSCs, adipose-derived stem cells; BEC, blood vascular endothelial cell; HDMEC, human dermal microvascular endothelial cell; IGF, insulin-like growth factor; LEC, lymphatic endothelial cell; LV, lymphatic vessels; HGF, hepatocyte growth factor; FGF, fibroblast growth factor.

Although LTE includes additional areas, as illustrated in Figure 3, this review focuses on those most relevant to LE.

### 5.1 Scaffolds and enhancing techniques

Scaffolds in lymphatic TE are designed for two main purposes: regeneration of LVs and regeneration of LNs (12). These scaffolds can be classified based on the type of biomaterial used in their fabrication. They include hydrogels [fibrin, nanofibrillar collagen, gelatin, matrigel, hyaluronic acid (HA), poly(ethylene glycol) (PEG), etc.,], decellularized ECM, biodegradable polymers such as polyglycolic acid (PGA), and non-biodegradable polymers including polyhedral oligomeric silsesquioxane poly (carbonate-urea) urethane (POSS-PCU), and silicone tubes (122, 123). Hydrogels are crosslinked polymer chains forming 3D network structures with high water content, a soft architecture, and high porosity, widely used in LVs generation (122, 132). A 3D HA-based hydrogel matrix, for example, was used to develop a platform with precisely controlled mechanical and biochemical cues, to modulate key lymphatic markers (Prox1, LYVE-1, and Pdpn), and to enable human LECs to self-assemble into functional LVs *in vitro* (133). In addition, HA-based hydrogels with high VEGF-C concentrations and tuned matrix stiffness promote the formation of lymphatic networks (134). A collagen derivative with orthogonal enzymatic crosslinking enables co-engineering of mature blood and lymphatic capillaries *in vitro* and *in vivo*, supporting vascularization without exogenous VEGF or supporting cells (135). It is important to highlight the role of hydrogels in the development of platforms for more precise studies of the angiogenesis process. For example, a PEG-based hydrogel platform has been developed to study LVs sprouting, providing a promising tool to understand the cellular mechanisms involved in LVs collateralization (136). Another important point to mention is that the function of the scaffold also depends on its properties, such as stiffness (137), specific strength, and flexibility (122). Another important point is that scaffold function depends not only on its intrinsic properties, such as stiffness (137), specific strength, and flexibility (122), but also on external mechanical cues, including interstitial flow and shockwave treatment, which increase cell permeability, enhance LECs proliferation, and support lymphangiogenesis both *in vitro* and *in vivo* (123, 129). Additionally, 3D bioprinting technology offers a means to create highly structured and customized scaffolds and to control cell deposition. Several bioprinting techniques have been developed, including extrusion-based printing, inkjet printing, and vat photopolymerization (138). Cell-laden scaffolds can be engineered, demonstrating lymphatic and vascular remodeling *in vivo* (139). Decellularization is another TE tool that can help remove all immunogenic components from tissue while preserving the ECM, which can be used in lymphatic regeneration. So far, this technique is more commonly used for LNs (140, 141). Thus, the development of strategies involving lymphatic regeneration is an imperative of the time and is oriented toward the creation of LVs, followed by their connection to systemic structures, the development of LNs and the vascularization of the LS (142).

Although scaffold-based strategies have shown encouraging results in preclinical studies, their clinical application in patients with LE remains limited. However, scaffolds are used in the clinic to support lymphatic drainage and lymphangiogenesis in patients with LE. Nanofibrillar collagen scaffolds have been successfully applied, in combination with other interventions, resulting in limb volume reduction and evidence of lymphangiogenesis on imaging. Silicone tubes have also been used to create artificial pathways for lymphatic drainage to the nearest LNs, showing a reduction in limb volume (143).

### 5.2 LN-focused tissue engineering

Different approaches have been used to replicate the structure and function of the LNs, including native tissue decellularized scaffolds, a variety of biomaterials, culture systems, and cell types to replicate stromal networks found *in vivo* (144, 145). Another possible solution for engineering LNs includes cryopreservation of stromal tissue, and it does not appear to significantly affect the regenerative function of node fragments (146). Recent studies address the potential use of decellularized LN as support for the treatment and prevention of LE. The study by Yang et al. demonstrated that after transplantation of decellularized LNs matrices in rats, functional integration with the host system and partial restoration of lymphatic drainage were achieved (140). Choosing an efficient method of decellularization of LNs to obtain scaffolds for TE is an important step that was studied in the work of Cuzzone in which it is demonstrated that decellularized LNs populated with cells are the basis of functional regeneration (141). A recent study in the field of lymphatic TE presents an optimized method for decellularization of LNs that helps preserve the native ECM after detergent application. The study also analyzes thin lymph node sections to ensure better nutrient penetration (147).

Kang et al. designed 3D bioprinted artificial LNs from alginate-atelocollagen hydrogels and adipose tissue mesenchymal stem cells. After transplantation of these scaffolds into rats in LE models, improvements in lymphatic drainage and regeneration of LVs, as well as reduction of edema, were observed. Histological examination showed the beginning of functional LNs formation (139). A recent work by Wenkai et al focuses on bioengineering strategies for creating capillaries, LNs by using different types of scaffolds, exploring the use of a synthetic lymphoid tissue organoid consisting of dendritic cells, stromal cells embedded in a collagen sponge and demonstrating that stromal cells can be applied in LNs engineering (116).

Unfortunately, LN-based TE remains experimental, and under our article selection criteria, no clinical trials evaluating engineered or decellularized LN constructs for LE treatment have been reported in the past 10 years.

Overall, *in vitro* and *in vivo* studies demonstrate that scaffold-based approaches can support lymphatic endothelial cell survival, migration, and organization into lymphatic networks. In particular, hydrogels, collagen-based matrices, and 3D-bioprinted constructs have been shown to promote lymphangiogenesis and improve lymphatic function in preclinical models. Lymph node–focused TE strategies, including decellularized, recellularized, and organoid-based constructs, have demonstrated the ability to restore lymphatic drainage and regeneration. However, these approaches remain largely at the preclinical stage, and further studies are required to evaluate their translational potential. The main *in vitro* and *in vivo* studies investigating these strategies are summarized in Table 5.

**TABLE 5:** Lymphatic tissue engineering: summary of *in vivo* and *in vitro* experiments.

Methods	Model	Approach and techniques	Outcome	Key citation
Scaffolds (148)				
PA hydrogels with tunable stiffness + cell culture (mouse LECs, mouse BC cells, human LECs).	*In vitro*.	ECM stiffness was used to study LEC biology, comparing normal tissue stiffness (1,500 Pa) and tumor microenvironment stiffness (10,000 Pa). Techs: Western blotting, wound healing, IF, and spheroid assays.	Matrix stiffening significantly promotes LEC proliferation and migration - enhanced proliferation was observed in both human and mouse LECs on stiff substrates (tumor-associated).	Xu et al. (137)
Modified gelatin hydrogels (GelTN) + HDLECs spheroids + (VEGF)-C.	*In vitro*; *in vivo* - mouse model.	Establishment of a 3D *in vitro* lymphangiogenesis model using GelTN gelatin hydrogels and testing GelTN injectability for controlled growth factor delivery *in vivo*. Techs: Viability (live/dead) assay, metabolic activity assay, microcarrier bead sprouting assay, LECs sprouting assay, confocal imaging, IF, H&E staining.	*In vitro*: HDLECs encapsulated in GelTN formed elongated lymphatic networks with significantly increased metabolic activity and sprouting within 14 days *in vivo*: cellular infiltration and perfused neo-vessel formation after 14 days after subcutaneous injection.	Al-Ansari et al. (149)
Synthetic HA hydrogels matrix + human LECs	*In vitro*; *in vivo* - mouse model.	To investigate the capability of a 3D HA-hydrogel synthetic matrix to generate LVs *in vitro* and *in vivo* in the absence of cellular support. Techs: IF, viability (live/dead) assay, perfusion assay with microbeads, real-time gene expression analysis, confocal imaging.	*In vitro*: Human LECs self-assembled into lymphatic tubes within 3 days and could be cultured for up to 3 weeks; *In vivo*: Engineered LVs continued to grow and integrated with host vasculature 2 weeks after implantation.	Alderfer et al. (133)
Collagen-based hydrogel chemically modified with enzyme-specific peptides.	*In vitro*; *in vivo* - rat model.	Was used orthogonal crosslinking approach to engineer a collagen-based hydrogel that supports controlled formation of blood and LVs in the absence of exogenous VEGF; Techs: IF (CD31, LYVE-1), spheroid outgrowth assay, confocal microscopy, histology, immunohistochemistry, mechanical testing.	*In vitro*: blood and lymphatic capillary networks began forming at day 7, with continued maturation by day 14; *in vivo*: subcutaneous implantation showed the anastomosis/inosculation of the implanted bioengineered capillaries with the host vascular network at day 7.	Rütsche et al. (135)
3D-bioprinted scaffolds (biomaterials + hADSCs). To prepare the bioink, sodium alginate and collagen were used.	*In vitro*; *in vivo* - rat model.	Potential of 3D-bioprinted scaffolds to promote lymphangiogenesis: The experimental groups were the control group (G1), hADSC group (G2), blank scaffold (G3), and hADSCs scaffold (G4) (*n* = 10 per group); Techs: Fluorescence imaging, histopathology, IF (LYVE-1 + Prox1, LYVE-1 + CD31), western blotting (VEGF-C, β-actin), quantitative polymerase chain reaction (mRNA levels of LYVE-1, VEGF-C, VEGF-A, and GAPDH), live/dead staining, mechanical testing, mid-thigh circumference, paw thickness in the hindlimbs, ICG.	3D-bioprinted scaffolds loaded with hADSCs showed good cell viability, maintained structural integrity, and enhanced lymphatic remodeling, promoting lymphangiogenesis and improving lymphatic function in LE.	Kang et al. (139)
Fibrin gel construct with LECs alone or combined with ASCs incorporated into an AV loop.	*In vivo* - rat model	To explore the role of constructs in lymphangiogenesis and their ability to bridge the lymphatic gap; Techs: Lymphatic markers: LYVE-1, podoplanin, VEGF-C, and VEGFR-3 analysis.	The level of lymphangiogenesis was significantly higher in the group with LECs alone. The engineered tissue restored lymphatic drainage and reduced local inflammation being transplanted into the area of LNs dissection.	Mackert et al. (150)
LN-focused tissue engineering				
Porcine dLNs	*In vivo* - mice model	dLNs transplantation to prevent hindlimb LE: subcutaneous dLNs implantation in LS-damaged rats vs. frozen porcine LNs implantation (sham group) vs. control group. The animals were sacrificed 2 and 4 weeks later (*n* = 18 per group); Techs: Hindlimb circumference, ICG, histology, IF & immunohistochemistry (CD3, CD20, CD4, LYVE-1), DAPI, DNA quantification.	At 12 POD, the control group exhibited the most severe limb swelling, whereas the dLN and sham groups showed milder edema; Neonatal LVs, immune cell infiltration, and the number of LYVE-1^+^ LVs were significantly higher in the dLN group.	Jian et al. (140)
Mice dLNs	*In vitro*; *in vivo* - mice model.	The antigenic potential of dLNs was tested by implantation into syngeneic and allogeneic mouse renal capsules (*n* = 4 per group) for 14 days; additionally, repopulated dLNs were seeded with leukocytes and implanted intramuscularly. Techs: DNA isolation, H&E, immunohistochemistry (CD45).	Efficient removal of nuclear material; preservation of structural integrity; absence of inflammatory reactions; successful delivery of immune cells *in vivo*.	Cuzzone et al. (141)
Lympho-organoids (LOs) obtained from LN stromal progenitor cells and dLN scaffolds.	*In vitro*; *in vivo* - mice model.	Therapeutic regeneration potential of LOs using *in vivo* models: four animal groups - control (*n* = 7), ALNT (*n* = 9), ALNT + dECM alone (*n* = 6), and ALNT + LOs (*n* = 13); Techs: DAPI, confocal analyses and IF, and immunohistochemistry (PROX-1, CD3, B220, Collagen IV, T and B cells), NIR imaging, CT-Scan.	Transplanted LOs integrated with host lymphatics, were repopulated by immune cells, and restored lymphatic drainage and immune function.	Lenti et al. (151)
Recellularized LN scaffold obtained from dLN + hADSCs.	*In vitro*; *in vivo* - rat model.	Effect of the designed scaffold on lymphangiogenesis in LE was evaluated in four study groups: control (*n* = 10), hADSC (*n* = 10), dLN scaffold (*n* = 10), and recellularized LN scaffold (*n* = 10); Techs: H&E, Masson’s trichrome staining, IF & western blot (VEGF-A, LYVE-1, β-Actin).	Recellularized LN scaffold demonstrated superior therapeutic efficacy compared to stem cells or dLN scaffolds alone, promoting stable lymphangiogenesis.	Kang et al. (152)

PA, Polyacrylamide; BC, breast cancer; LECs, lymphatic endothelial cells; HDLECs, human dermal LECs; IF, immunofluorescence; AV, arteriovenous; ECM, extracellular matrix; (VEGF)-C, vascular endothelial growth factor; GelTN, tetrazine and norbornene modified gelatin hydrogels; H&E, Hematoxylin & Eosin; HA, hyaluronic acid; dLN, decellularized lymph node; POD, postoperative day; hADSCs, human adipose-derived stem cells; GAPDH, glyceraldehyde-3 phosphate dehydrogenase; VLNT, Vascularized autologous lymph node transfer.

### 5.3 Lymphangiogenesis

LVs develop during embryogenesis from the cardiovascular system, at the end of the fifth week of human embryonic development (153). Vascular endothelial cells begin expressing LYVE-1, and transcription factors SOX18 and COUP-TFII activate PROX1, the key regulator of lymphatic identity. PROX1 increases VEGFR-3 expression, making cells more responsive to VEGF-C and promoting LECs polarization and lymph sac formation (86). LEC progenitors expressing the transcription factor PROX1 begin to migrate from the cardinal vein, giving rise to the first primitive LVs, known as the dorsal peripheral longitudinal LVs, in the jugular region of the embryo. The second population of cells forms the ventral primordial thoracic duct, which expands further by lymphangiogenic sprouting (154). In adults lymphangiogenesis occurs physiologically during wound healing, tumor metastasis, and transplant rejection (121). During wound healing, lymphangiogenesis may occur through either self-organization of migrating LECs or sprouting from pre-existing lymphatic capillaries (153). Multiple signaling pathways and molecular factors play essential roles in regulating proper LVs formation (122). However, the molecular signaling mechanism of lymphangiogenesis is primarily regulated by the VEGF-C/VEGFR-3 signaling axis, which acts downstream of PROX1-mediated lymphatic specification and coordinates lymphatic endothelial cell proliferation, migration, and vessel maturation through PI3K/AKT and MAPK/ERK pathways. Additional regulation is provided by angiopoietins, fibroblast GFs, vascular endothelial GFs C and D, hepatocyte growth factor, inflammatory cytokines, and ECM-mediated signaling (153–155).

Identification of lymphatic-specific genes and proteins has advanced the understanding of lymphangiogenesis and supported the development of novel therapeutic strategies for LE (116). LTE targets lymphangiogenesis primarily through the use of cells (156), GFs (157), and gene therapy (123). In combination with additional approaches such as scaffolds and *in vitro* and *in vivo* models, these strategies aim to promote the development of functional tubular lymphatic networks (122).

#### 5.3.1 Cells

For a long time, the only options for isolating LECs were the dermis, intestine, and LNs; however, the differentiation of induced pluripotent stem cells (iPSCs) into the lymphatic lineage has become a promising option (123), and also the use of lymphatic-specific antibodies to isolate primary LECs represents a major advance (158). Gibot et al. (159) describe a five-step protocol for the *in vitro* organization of human dermal LECs into a stable 3D lymphatic capillary network, co-cultured with dermal fibroblasts, without using scaffolds or GFs (159). Park et al. (160) discussed the role of bone marrow–derived lymphatic endothelial progenitor cells in lymphatic neovascularization (160). Later, Ismail et al. (161) reported the results of a randomized controlled clinical trial demonstrating the effectiveness of bone marrow-derived mononuclear cells in the treatment of patients with primary lower limb LE. The study showed that cell therapy can improve limb circumference, relieve pain, and enhance walking ability (161). More recently, Miorita et al. (162) demonstrated the efficacy of neonatal porcine bone marrow–derived mesenchymal stem cell (NpBM-MSC) xenotransplantation for the therapy of hind limb LE in mice. The results showed that NpBM-MSCs secrete angiogenic and lymphangiogenic factors and increase the expression of porcine VEGF genes and TGFB1 at the transplantation sites, until they were rejected at POD 14 (162). ADSCs have also been demonstrated to have the ability to differentiate into various cell lineages, including endothelial cells (ECs) (163), and studies further indicate their ability to differentiate into LECs and secrete lymphangiogenic GFs (164–166). Ahmadzadeh et al. (67) demonstrated the effectiveness of GFs secreted by human ADSCs in stimulating human dermal LECs (HDLECs) *in vitro*, showing a stronger effect than individual GFs applied alone (167). Additional studies have shown that engineered ADSCs and ADSC-derived exosomes both improve edema and subcutaneous fibrosis in a mouse model of secondary LE (168, 169). It has also been reported that platelet-rich plasma significantly increases lymphangiogenesis during the wound-healing process (170).

#### 5.3.2 GFs and genes

Regarding the GFs used in lymphatic TE to promote lymphangiogenesis, VEGF-C and VEGF-D play a central role by binding to the endothelial cell–specific tyrosine kinase receptors VEGFR-2 and VEGFR-3, which are crucial regulators of angiogenesis and lymphangiogenesis (117). However, both VEGF-C and VEGF-D are also capable of activating VEGFR-2, the principal mediator of angiogenesis. To achieve selective stimulation of lymphangiogenesis through VEGFR-3, a point-mutated form of VEGF-C (VEGF-C156S) was developed. This variant exhibits enhanced specificity for VEGFR-3 and has gained considerable interest as a targeted therapeutic approach for LE. Nonetheless, Forte et al. (171), in a systematic review providing a comprehensive synthesis of the available evidence on VEGF-C156S, reported that native VEGF-C appears to exert a stronger lymphangiogenic potential compared with the mutated form (157). Furthermore, comparative experimental evidence indicates that VEGF-D gene expression obtains a weaker lymphangiogenic response than VEGF-C, suggesting a comparatively lower potency in stimulating lymphatic vessel formation (171). Nevertheless, VEGF-C–induced lymphangiogenesis depends not only on VEGF-C/VEGFR-3 signaling but also on the Angiopoietin (Ang)–Tie receptor pathway and downstream PI3K signaling. Korhonen et al. (172) showed that Ang2 and its receptors, Tie1 and Tie2, are required to maintain VEGFR-3 on the surface of LECs, and that disrupting Ang2, Tie receptors, or PI3K signaling reduces cell-surface VEGFR-3, leading to impaired lymphangiogenesis in mice (172). Also, the VEGF-C/VEGFR-3 axis is widely proposed as a high-potential target to promote lymphatic capillary formation; additionally, ANGPT1/2/TIE2 signaling could guide postnatal maturation of LVs, and the ALK1 pathway is of interest due to its role in regulating the differentiation of immature LECs into mature LECs (117, 173).

## 6 Potential translational applications

Currently, the only therapeutic options for patients with LE are complete decongestive therapy and microsurgery. Decongestive treatments provide only transient relief and must be maintained lifelong. At the same time, surgical methods such as LVA and VLNT have specific limitations in their indications. These include the requirement for suitable LVs for anastomosis and the risk of complications, such as iatrogenic LE at the donor site (10, 117). In this context, LTE may represent a promising complementary approach to current microsurgical treatments for the management of LE (10).

Based on the literature review, clinical translation is more challenging for lymph node–based therapies because of their complex microarchitecture and incomplete mechanistic understanding (10). Meanwhile, models of LVs still require refinement to better replicate button-like cell-cell junction formation, valve function, and lymphatic muscle cell contraction, thereby enabling the reproduction of functional lymphatics (11).

However, it is important to emphasize the progress in the field of lymphatic TE; collagen-based scaffolds have demonstrated efficacy due to their biocompatible and biodegradable properties (122). Yesantharao and Nguyen (174) discuss the efficacy of nanofibrillar collagen scaffolds in both preclinical and clinical contexts in a review article based on the analysis of 32 relevant studies. In summary of clinical investigations, it has been shown that this type of scaffold, used in combination with surgical methods, has a good outcome in limb volume reduction, improved neuropathic pain, reduced infections, and improved patient satisfaction (174). At this point, it is important to mention the vital relationship between LECs and the ECM microenvironment. Thus, the knowledge regarding ECM molecules can provide critical prerequisites for LEC growth, migration, tube formation, and survival during lymphangiogenesis. Furthermore, understanding the signaling pathways involved in lymphangiogenesis may be helpful in identifying novel therapeutic targets and improving strategies for lymphatic regeneration (158). Among the advances in this direction are fully defined 3D synthetic matrix systems capable of generating LVs *in vitro* (133), regulation of matrix stiffness and controlled delivery of GFs (VEGF-C) on directing lymphatic tube formation (134), the development of *in vivo* models for functional LVs formation (175). Stimulating lymphangiogenesis can promote tissue homeostasis, wound healing, reduce inflammation, and ultimately enhance tissue repair and regeneration in a variety of organs. Insufficient lymphangiogenesis after ischemia can lead to reduced function of the LVs. In these circumstances, inducing lymphangiogenesis using GFs (VEGF-C/D) or activating VEGFR3 can be beneficial for supporting tissue repair and regeneration (176). Bioactive scaffolds containing lymphangiogenic-specific signals may support lymphatic growth and enhance treatment outcomes in both primary and secondary LE (117). Also, GFs, cells, and mechanical stimulation have been shown to be effective in the reconstruction of lymphatic vasculature at multiple scales, including lymphatic capillaries, collecting LVs, and LNs (12).

It is also relevant to highlight advances in LNs-based TE, including *in vivo* models for LNs-like structure formation (126), artificial LNs matrix (177), decellularized LNs scaffolds (141), and recellularized LN ECM (152). In recent years, key regulators of lymphangiogenesis have been elucidated and studied. With the growing ability to develop new techniques in biomaterials, cell culture, and imaging, as well as advances in cells and signaling pathways, this knowledge may contribute to the successful development treatments for different forms of LE (173). These applications are not limited to patients with affected lymphatic organs; it may also be possible to utilize lymphatic TE in concert with other surgical interventions to reduce the risk of immune rejection of transplanted allografts or engineered tissues. Additionally, there is growing interest in developing artificial lymphatic organs as a potential therapeutic strategy in oncology (120).

Despite advances in LTE, most approaches remain at the preclinical stage, and their translation into clinical trials may be delayed by general limitations in TE, including cell selection, inhomogeneous cell distribution, cell adhesion and retention, scaffold mechanical integrity and stability, and immunogenicity (119).

## 7 Conclusion

As a progressive disease, early diagnosis and treatment of LE are crucial. Despite advances in understanding its pathophysiology, LE remains an incurable condition that requires lifelong management. Currently, available treatment options primarily focus on symptom control rather than targeting the underlying disease mechanisms.

Advances in TE highlight key areas, including biomaterials, lymphangiogenesis, LN-based TE, and a growing understanding of lymphatic regenerative biology. These fields offer distinct yet complementary paths for future advances. Scaffold-based methods are increasingly designed to create supportive microenvironments that foster lymphatic regeneration and integration. LN-based TE focuses on restoring functional LN units through structural reconstruction or *de novo* formation. Meanwhile, strategies targeting lymphangiogenesis aim to stimulate the growth and remodeling of lymphatic networks. Future therapeutic approaches for LE are expected to shift from symptomatic management toward targeting the underlying etiopathogenesis of the disease.

Despite the numerous advantages of LTE, as with all branches of regenerative medicine, researchers must still address common challenges to achieve clinical application, including cell sourcing and selection, cell survival and integration, establishment of a functional vascular network, management of immunogenicity, and ensuring long-term structural integrity, functionality, and maintenance of the desired phenotype *in vivo*.

Although LTE strategies remain largely experimental and continue to evolve, they represent a promising and important future direction for the treatment of LE.
