# Supplementary material for: Lymphedema: current treatment strategies and future directions in tissue engineering
Source: Front Med (Lausanne). 2026 Aug 5;13:1851346. doi: 10.3389/fmed.2026.1851346 (PMC13487752; doi:10.3389/fmed.2026.1851346)
Supplement: Supplementary file 1 [file Table_1.docx]

Table 1. **Workflow of literature identification and selection**

| **Literature Selection Process** | **Description** |
| --- | --- |
| Databases | PubMed, Scopus, and Google Scholar |
| Language criteria | English, Hungarian, German, and French |
| Keywords used (combinations) | Disease terms   1. Lymphedema 2. Primary lymphedema 3. Secondary lymphedema   Anatomy   1. Lymphatic system 2. Lymphatic vessels 3. Lymph nodes 4. Lymphangiogenesis   Pathophysiology   1. Etiopathogenesis 2. Pathophysiology 3. Inflammation 4. Fibrosis   Treatment   1. Treatment 2. Therapy 3. Surgical treatment 4. Conservative treatment   Tissue engineering   1. Lymphatic tissue engineering 2. Regenerative medicine 3. Cell therapy 4. Stem cells 5. Biomaterials 6. Lymphatic reconstruction 7. Large/small animal model |
| Selection criteria | - Open-access, peer-reviewed articles and book chapters; - Publications relevant to the etiology, pathogenesis, diagnosis, treatment, and regenerative approaches to lymphedema; - Original research articles, clinical studies, experimental studies, review articles, and book chapters; - Full-text publications available in English, Hungarian, German, and French. |
| Exclusion criteria | - Publications not directly related to lymphedema; - Articles lacking relevance to the topics of etiology, diagnosis, treatment, or lymphatic tissue engineering; - Editorials, letters to the editor, and commentaries; - Duplicate publications; - Publications with inaccessible full text; - Publications in languages other than English, Hungarian, German, and French. |
| Final literature | 177 articles were included in the review |
